# Understanding how and why de-implementation works in health and care: research protocol for a realist synthesis of evidence

**DOI:** 10.1186/s13643-019-1111-8

**Published:** 2019-08-05

**Authors:** Christopher Burton, Lynne Williams, Tracey Bucknall, Stephen Edwards, Denise Fisher, Beth Hall, Gill Harris, Peter Jones, Matthew Makin, Anne McBride, Rachel Meacock, John Parkinson, Jo Rycroft-Malone, Justin Waring

**Affiliations:** 1 Noreen Edwards Chair of Rehabilitation and Nursing Research, Head of School, School of Health Sciences, Bangor University, Bangor, Gwynedd UK; 2 Bangor University, Bangor, UK; 3 School of Nursing and Midwifery, Deakin University, Melbourne, Australia; 4 Betsi Cadwaladr University Health Board, Bangor, UK; 5 The Pennine Acute Hospitals NHS Trust, Greater Manchester, UK; 6 Alliance Manchester Business School, Manchester University, Manchester, UK; 7 Division of Population Health, Health Services Research and Primary Care, Manchester University, Manchester, UK; 8 Nottingham University Business School, Nottingham University, Nottingham, UK

**Keywords:** De-implementation, Low-value practice, Overuse, Health services, Concept analysis, Realist synthesis

## Abstract

**Background:**

Strategies to improve the effectiveness and quality of health and care have predominantly emphasised the implementation of new research and evidence into service organisation and delivery. A parallel, but less understood issue is how clinicians and service leaders stop existing practices and interventions that are no longer evidence based, where new evidence supersedes old evidence, or interventions are replaced with those that are more cost effective. The aim of this evidence synthesis is to produce meaningful programme theory and practical guidance for policy makers, managers and clinicians to understand how and why de-implementation processes and procedures can work.

**Methods and analysis:**

The synthesis will examine the attributes or characteristics that constitute the concept of de-implementation. The research team will then draw on the principles of realist inquiry to provide an explanatory account of how, in what context and for whom to explain the successful processes and impacts of de-implementation. The review will be conducted in four phases over 18 months. Phase 1: develop a framework to map the preliminary programme theories through an initial scoping of the literature and consultation with key stakeholders. Phase 2: systematic searches of the evidence to develop the theories identified in phase 1. Phase 3: validation and refinement of programme theories through stakeholder interviews. Phase 4: formulating actionable recommendations for managers, commissioners and service leaders about what works through different approaches to de-implementation.

**Discussion:**

This evidence synthesis will address gaps in knowledge about de-implementation across health and care services and ensure that guidance about strategies and approaches accounts for contextual factors, which may be operating at different organisational and decision-making levels. Through the development of the programme theory, which explains what works, how and under which circumstances, findings from the evidence synthesis will support managers and service leaders to make measured decisions about de-implementation.

**Systematic review registration:**

PROSPERO CRD42017081030

## Background

In the current climate, health and care systems are under pressure to demonstrate that actions to ensure efficient use of resources do not compromise the quality of the care provided. However, practices that have limited evidence for their efficacy continue to be performed, for example, imaging for low back pain, antibiotics for sinusitis, and antipsychotics for dementia [1]. In the USA, it has been estimated that 30% of medical spending is considered to be unnecessary [2], and a report in 2010 estimated the cost of prescribed medicines wasted in the UK to be around £300 million each year [3]. Globally, there is growing emphasis placed on prudent and efficient use of resources for all health and care services [4–6].

De-implementation is one of many terms used in the literature to describe the removal or reduction of costly or potentially hazardous approaches to care. A scoping review [7] found 43 descriptors, including, for example, disinvest, de-adoption, discontinue, withdraw and de-commission. De-implementation is defined as “stopping practices that are not evidence-based” [8]. There may be an assumption that de-implementation is the reverse process of implementation, and as such, there is some transferability of the theory and frameworks between the two. However, the factors that shape the processes of implementation and de-implementation are likely to be different and work in different ways [8]. For example, a mixed-methods study of biomarker blood tests for breast cancer surveillance within an integrated health care system [9] found that the usual strategies for implementation, such as improving awareness and knowledge, were unlikely to be effective for de-implementation. Government policy in high-income economies highlights the importance of health and care delivery which is efficient and prudent and makes best use of resources where these may be limited. These include the ‘Choosing Wisely’ campaign in Canada, the USA and Australia [5, 10]. Other examples include ‘Realistic Medicine’ [11], which promotes treatment that is focused on the probability of benefit rather than the possibility of benefit, and ‘Prudent Healthcare’ [6], which discourages the tendency to intervene where a lesser intervention might suffice.

There are some individual studies that have specifically paid attention to de-implementation. For example, a study that explored the literature and analysed data about implementation and de-implementation from two studies among Dutch orthopaedic surgeons concluded that further research is required to understand the required leadership and champion characteristics that can accelerate the process of de-implementation [2]. The authors also call for more research to establish the factors which influence the success or not of de-implementation, and better understanding of the range of de-implementation strategies. A recent scoping review found many different terms relating to the process, but crucially identified several questions for further research [7]. These included the need to disentangle the practices which may be ineffective for one population group but effective for another and better understanding of the factors which determine why practices should be de-implemented or de-adopted. Barriers to de-implementation are numerous and complex, and overcoming these require an over-reaching approach at different levels to support patients, clinicians and the system [9].

In the drive to increase health service efficiency, current health and care policy and guidance highlight the importance of using only clinical and other practices which are effective and valuable and reflect the high quality of care for patients. Consequently, interest is developing in evidence to inform health professionals about de-implementation [7–9]. There are a plethora of challenges for managers and service leaders that are intent on delivering efficient services, including managing budgetary constraints alongside professional obligations and patient expectations; commissioning of services; media and public concerns about service quality; and the drive for innovation. There is often insufficient consideration about how to replace existing practices and service pathways. Despite reflecting a renewed dedication to evidence-based healthcare [9], researchers have highlighted that disinvestment for commissioners and managers lacks guidance [12]. The growing interest in de-implementation and disinvestment in ineffective practices is therefore timely and important. The provision of evidence-based guidance, underpinned by robust programme theory, has the potential to support managers and service leaders to improve service efficiency and quality.

Realist synthesis or review is a systematic, iterative, theory-driven approach that draws on a heterogeneous evidence base to establish what works, how, in what context and for whom [13]. Unlike systematic reviews, realist syntheses use key stakeholders’ own theories alongside the literature to elicit and test programme theories that can be applied. Additionally, realist syntheses draw on different theoretical and disciplinary perspectives that enable the generation of new insights, which in this case may influence de-implementation.

## Research question and aims

The principal aim of this realist synthesis is to generate evidence and theory to guide managers and service leaders to effectively de-implement practices across health and care services. The output generated will be in the form of a programme theory, populated with evidence that describes what works about de-implementation, how and the contextual factors that influence their impact on outcomes. We will collaborate with managers and health and care leaders to translate this programme theory into actionable recommendations for different organisations, populated with practical examples from the evidence.

The main aims are:

1. To identify and map the range of different de-implementation approaches and/or strategies currently being utilised across health and care and in the fields of behavioural psychology, behavioural economics, organisational sociology, crime and deviance, paying attention to ways in which they are assumed to work
2. Produce a typology of de-implementation types, processes, contexts
3. Examine and understand the range of impacts of these approaches and/or strategies across different settings, paying attention to contextual conditions which influence how they work
4. Produce an evidence-based, realist programme theory that explains the successful processes and impacts of de-implementation
5. Explore, through stakeholder engagement, the decision-making processes associated with de-implementation
6. Provide recommendations about ways in which different approaches and/or strategies can help managers and service leaders plan and prioritise de-implementation in a systematic and efficient manner
7. Stimulate a wider debate about avoiding and stopping services that are considered wasteful, of low value and non-efficient, for future provision

## Methods

Drawing on our previous experience of conducting realist syntheses (e.g. NIHR HS&DR 12/129/32: HS&DR 14/194/20), and publication guidance for realist synthesis [14], the work will be conducted over four phases. Utilising the realist approach will enable the development, testing and refinement of a programme theory about de-implementation, which considers the recognised individual, social and organisational complexities that underpin health and care systems. The main output generated will be in the form of a programme theory, populated with evidence that describes ‘what works’ about de-implementation and focuses on the contextual factors that influence their impact on outcomes. We will work with NHS managers to translate this programme theory into actionable recommendations populated with practical examples from the evidence. The evidence synthesis will be completed within 18 months.

The protocol was registered with the International Prospective Register of Systematic Reviews (PROSPERO) on November 17, 2017 (registration number CRD42017081030). The protocol has been reported according to the Preferred Reporting Items for Systematic Reviews and Meta-analysis Protocols (PRISMA-P) 2015 checklist [15].

### Research plan

#### Phase 1: Programme theory development

The first phase involves the development of the initial programme theory, a practical framework which represents what is thought to work, how and under which conditions, about successful de-implementation practices that are of low value and not evidence-based. This work will draw on an extensive scoping review of the existing literature and theories. We will incorporate consultation with stakeholders (i.e. managers and organisational leaders drawn from the project team networks) and patient and public involvement (PPI) representatives through workshops. The purpose of the workshops is to develop an understanding of the complexities of the contexts in which de-implementation efforts are situated. Through interactive workshops, participants’ thoughts and ideas about de-implementation can be made visible, which serves as a basis for discussion to find out the contextual influences within systems that might impact on different de-implementation strategies.

To build on the theory-building work, we will additionally conduct telephone interviews with clinicians, managers and organisational leaders. A semi-structured interview schedule will be developed using the emerging theories from the workshops and will focus on understanding the decision-making processes and influences on participants’ judgements during de-implementation processes. Using the principles of framework analysis [16], we will integrate the workshops’ findings with the telephone interview data.

Together, these activities will contribute to the development of an initial programme theory that provides an early explanation of the complexity of de-implementation and the contextual conditions that underpin the process. The initial theoretical framework will guide the literature mining and consultation work with stakeholders. To initiate the process, we will draw on different theories and approaches, some of which may traditionally be used in investigations of ‘positive’ implementation. Elements of these theories will provide the conceptual platform for commencing phase 1 of this synthesis. These may include habit formation, unlearning, decision-making theories, nudge and behaviour change, organisational sociology and diffusion of innovation. It is the initial programme theory that is tested in the following phases.

#### Phase 2: Evidence retrieval, data extraction and synthesis

In phase 2, we will draw on evidence to test and refine the programme theory. In the spirit of the realist approach, priorities are always focused on theory development rather the internal validity of the evidence, and a diversity of evidence provides more opportunities for mining the evidence and seeking an in-depth explanation. As some evidence about de-implementation is likely to be unpublished, we will use our stakeholder group and wider networks across different organisations to identify a range of possible evidence sources. Inclusion of grey literature, e.g. in the form of project reports, case studies, campaigns, policy evaluation, film clips and secondary data, will ensure that important evidence from a range of perspectives is included. The review process includes screening literature for evidence of relevance and extracting and charting key data around the programme theory.

Our search will not be limited to health and care, to ensure classic studies on social group dynamics and behaviour change can be included, but specific literature and evidence published in the last 20 years will capture the emergence of the ‘what matters is what works’ philosophy [17] and international policy campaigns to improve efficiency in health and care services [5]. All materials will be managed in Mendeley (an online bibliographic management programme). We will include material indexed in the major health and related databases, to include Cochrane Library, Campbell Collaboration, MEDLINE, CINAHL, NIHR Journals Library, IBSS, HMIC, ASSIA, Sociological Abstracts, Social Policy and Practice and Social Care Online. The search for references will additionally be concerned with non-health-related domains to identify successful de-implementation approaches that might have value when applied within a health context.

### Search terms

We will determine keywords starting with the 43 terms listed by Niven et al. [7] in a scoping review of de-implementation and choose the terms that are listed as being most often present in the citations they are located. We have undertaken a test title only search in CINAHL and MEDLINE limited to human and English language using: TI (disinvest* OR dis-invest* OR “decrease use” OR discontinu* OR dis-continu* OR abandon* OR reassess* OR re-assess* OR obsole* OR “medical reversal” OR contradict* OR withdraw* OR “health technology re-assessment” OR deimplement* OR de-implement*) N3 (healthcare or technolog* or device* or intervention* or health practi?e* or medical or medical practi?e* or procedur* or drug or drugs or biotechnology*) and found 100 results (CINAHL) and MEDLINE 1123. We screened 10% of these and found 55 titles that appear initially of interest to the synthesis.

### Other searches

We will also conduct Internet-based searches for grey literature, such as de-implementation reports relating to national and local initiatives; where possible, evaluative information about these initiatives held in the public domain will be requested. We will also use citation tracking and snowballing techniques and draw on the expertise of the project team, steering group, other key researchers and service leaders to ensure we have not missed evidence that might be relevant, but not visible through traditional and hand searching methods.

### Grey literature

We will draw on the experience and networks of our project team in the identification of interventions and programmes that describe de-implementation. We will scope Internet resources and identify repositories of research and relevant reports, to seek relevant guidance, for example, NHS evidence (https://www.evidence.nhs.uk/) and Open Grey (http://www.opengrey.eu/).

### Inclusion and exclusion criteria

We will use purposive sampling to test and refine the programme theories from phase 1. This ensures an inclusive approach to the search process, recognising that different types of research, policy, guidance and reports can be used in order to find and evaluate the evidence to test and refine the programme theories.

### Review and extraction

Screening for relevance to the programme theories and data will involve a systematic approach, in a system developed in a previous funded realist study (NIHR HS&DR projects 12/129/32). Evidence will not be excluded, unless it does not relate to the programme theory areas. The programme theories being ‘tested’ through the review are made visible through bespoke data extraction forms [18]. The test for inclusion will be if we consider that the evidence is ‘good and relevant enough’ to be included [13]. We will use a flow chart to support the judgement made of the extracted data and reported its potential to contribute to the review. The project team will undertake member checking in the reviewing and extracting processes, and any discrepancy in opinions about the relevance of evidence will be resolved through discussion amongst the project team.

### Synthesis

The analytical task involves synthesising across all extracted information to search for the relationships between mechanisms, contexts and outcomes. Through our previous experience of realist synthesis (NIHR HS&DR projects 12/129/32:14/194/20) [19, 20] and building on the suggestions of Pawson [13] and principles of realist enquiry, we will:

1. Organise the extracted information into data tables representing the different bodies of evidence that inform the programme theory areas, and the range of potential impacts from different de-implementation strategies and approaches (e.g. greater awareness, knowledge and understanding, change in attitudes and perceptions or direct practice change).
2. Undertake an abductive and retroductive approach [21] to look for and understand the best explanation of the cause in the evidence and look for demi-regularities (recurring patterns) in the Context (C) Mechanism (M) and Outcomes (O).
3. Link the demi-regularities to form a refined programme theory, consisting of CMOs which are explanatory statements reflecting the complexity of de-implementation strategies and processes. This explanatory account will provide an explanation of (for de-implementation across health and care services) what works, how and under which circumstances.

#### Phase 3

To test and refine the programme theory, phase 3 work will provide a check of validity of the findings from the literature in relation to ‘what works,’ how and under which conditions in the current approaches to de-implementation of health and care practice and interventions. In 10 audio-taped telephone interviews with managers and organisational leaders, we will analyse data to test the synthesis findings and refine the programme theory, to specify its practical relevance/potential. As in phase 1, we will use both purposive and convenience sampling strategies to identify clinicians, managers and organisational leaders who can help us develop the realist syntheses programme theory. A semi-structured interview schedule will be developed that reflects the constituents and links between elements of the programme theory, and the interviews will focus on ‘sense-checking’ from the participants’ perspectives. We will use thematic analysis to analyse the data.

##### Outputs from phase 3

- A refined set of hypotheses with accompanying evidence-based narrative.

#### Phase 4

In the final phase of the study, we will develop actionable recommendations for managers and service leaders. We will convene a second workshop with key stakeholders, which will allow us to test out and refine any element of the programme theory. We will also engage with the workshop participants and our broader network of stakeholders to (a) ensure different approaches to capture the right audience are used, (b) check that we are using language appropriate information for different audiences and (c) ensure that the key points from the synthesis are conveyed effectively for managers and service leaders, patients and the public.

## Project outputs and dissemination

Using our synthesis findings, we will recommend a series of strategies that will help managers and clinicians de-implement low-value processes, and disseminate these recommendations through the following channels:

- A full final report, using the evidence to provide examples of approaches and strategies to de-implementation, what features promote their use and success, what hinders them, and an understanding of the contextual influences which enable or disable their use
- An executive summary of the final report, suitable for use as a separate report
- A plain English summary of the report, suitable for use for briefing patients and the public
- Open-access publications of findings that set out an implementation plan for de-implementation.
- A range of blog posts to engage different audiences with the study and the findings
- Presence and presentation at a nominated UK conference, which is focused on the strategies to improve efficiency and value in health and care
- Creative animation easily accessible on YouTube
- A comprehensive evidence digest of our findings that provides an information selection source of the latest evidence about de-implementation
- We will engage with teams of other commissioned studies to share learning and expertise around de-implementation.

## Discussion

This evidence synthesis is important for the public and patients, and the NHS as the current use of ineffective practices that lack an evidence-base or value translate into sub-optimal care and a waste of resources. Additionally, as an increasing number of new innovations are developed, there is a need to understand how these could replace or modify (rather than add to) existing practices and services. This evidence synthesis will address gaps in knowledge about de-implementation across health and care services and ensure that guidance about strategies and approaches takes into account contextual factors, which might be operating at different organisational and decision-making levels. To reflect the ethos of the realist approach, attention will be paid to the constituents of de-implementation strategies and approaches across different health and care organisations, and in articulating the explanation of how particular features of these approaches are more likely to promote efficiency and high-quality care for patients. Through the development of the programme theory, which explains what works, how and under which circumstances, findings from the evidence synthesis will support managers and service leaders to make the right decisions about de-implementation, whilst concurrently promoting the confidence of patients and the public and generating the potential for efficiencies.

## Data Availability

The datasets used will be available from the corresponding author on reasonable request.

## Abbreviations

ASSIA: Applied Social Sciences Index and Abstracts
CINAHL: Cumulative Index of Nursing and Allied Health Literature
CMOs: Context-Mechanism-Outcomes
HMIC: Health Management Information Consortium
HS&DR: Health Services and Delivery Research
IBSS: International Bibliography of the Social Sciences
NHS: National Health Service
NIHR: National Institute for Health Research
PPI: Patient and public involvement
PROSPERO: Prospective Register of Systematic Reviews
REC: Research Ethics Committee
UK: United Kingdom
